# The social process of escalation: a promising focus for crisis management research

**DOI:** 10.1186/1472-6963-12-161

**Published:** 2012-06-15

**Authors:** Johan Bergström, Sidney Dekker, James M Nyce, Isis Amer-Wåhlin

**Affiliations:** 1 Lund University Centre for Risk Assessment and Management, PO-Box 118, SE-22100, Lund, Sweden; 2 Griffith University, School of Humanities, Brisbane, Australia; 3 Ball State University, Department of Anthropology, Muncie, IN, USA; 4 Karolinska Institute, Department of Women and Child Health, Stockholm, Sweden

## Abstract

**Background:**

This study identifies a promising, new focus for the crisis management research in the health care domain. After reviewing the literature on health care crisis management, there seems to be a knowledge-gap regarding organisational change and adaption, especially when health care situations goes from normal, to non-normal, to pathological and further into a state of emergency or crisis.

**Discussion:**

Based on studies of escalating situations in obstetric care it is suggested that two theoretical perspectives (contingency theory and the idea of failure as a result of incomplete interaction) tend to simplify the issue of escalation rather than attend to its complexities (including the various power relations among the stakeholders involved). However studying the process of escalation as inherently complex and social allows us to see the definition of a situation as normal or non-normal as an exercise of power in itself, rather than representing a putatively correct response to a particular emergency.

**Implications:**

The concept of escalation, when treated this way, can help us further the analysis of clinical and institutional acts and competence. It can also turn our attention to some important elements in a class of social phenomenon, crises and emergencies, that so far have not received the attention they deserve. Focusing on organisational choreography, that interplay of potential factors such as power, professional identity, organisational accountability, and experience, is not only a promising focus for future naturalistic research but also for developing more pragmatic strategies that can enhance organisational coordination and response in complex events.

## Background

This study will outline what has been identified as a promising focus of future research into health care crisis. Reviewing the research of health care crisis management, there seems to be a knowledge-gap concerning the process of organisational change and adaption, especially as a situation goes from normal, to non-normal and then further into a state of crisis. Following the identification of a knowledge-gap an outline of a research agenda will be presented, intended to support the study of the process of escalation in health care.

### Crisis

There is a growing body of research that looks at crisis (or emergency) management in different health care settings. Defined by Runciman [1] a crisis is “the point in the course of a disease at which a decisive change occurs leading either to recovery or to death” (p. 156). The definition treats the notion of crisis in binary terms as a clear transition from a non-crisis state to a state of crisis. In the same way crisis management is defined as the efforts to recover from this loss of control [2]. In other words, crisis management is seen as the process of returning to the previous binary state or status. This process of recovery from a loss of control is typically dealt with by means of crisis management training. Team training concepts, with their origin in industries such as aviation [3], have been introduced into healthcare with the aim of enhancing non-technical skills such as leadership [4], situation awareness [5], communication, and decision making [6,7]. Team training typically is performed in simulated environments [8,9] and researchers have put much effort into the development of these environments and methods to assess the effects of such training. However these assessment cycles tend to be tautological in that they only measure a certain set of crisis management skills like improved teamwork processes by ensuring certain “correct behaviours” of individuals [10]. There is also a debate whether enhancing these non-technical skills have the payoff those who advocate instruction and remediation of these kinds believe they actually have [11-14].

Previous research into health care crises acknowledge that the process(es) at the centre of any crisis (like the conditions and symptoms of the human whose life is in danger) can be complex, reflecting any number of sometimes even opposing goals as well as high uncertainty. Still the literature tends to treat organisation boundaries and the organisation itself as stable entities. However in any modern hospital different sub-organisations like the labour ward, the operation ward and the paediatrics ward are typically loosely coupled (with a high degree of self-governance and local adaption) to each other. In crisis situations however they can become tightly coupled and highly interdependent (e.g. when performing a Caesarean section). This can lead to new, emerging and inherently complex properties of the system as a whole – ones that can be temporary or permanent or both [15].^a^ But when does this tight coupling occur? What characterises this coupling? Which actors become tightly coupled and why?

The questions asked offer a promising way forward for studying medical crises. Typical solutions to the problem of managing crises have taken the form of proposals to improve behaviour (as described above) and implementation of more rigid workplace routines and structures. However from a complexity perspective, such strategies seem to be problematic. Rather than focusing on individual behaviour, the complexity perspective turns instead to the notion of bounded (or local) rationality [16]. Because certain behaviour can seem irrational or even erroneous from one perspective (particularly in the aftermath of an incident) does not necessarily mean that it was irrational or erroneous at the time of the incident [17], making notions such as “violations” highly problematic [18]. When it comes to the implementation of protocols, rules and routines as solutions to issues of health care safety, the complexity perspective counsels caution and suggests there may be serious limits to epistemology that underlies them and consequently the kinds of solutions that so far emerged [19-22].

While these issues have not been extensively studied by medical researchers, scholars interested in naturalistic settings of joint cognitive systems have given them some attention [23]. Some examples of studies of health care complexity includes studies of safety under dynamic and changing conditions [24], the way in which technology, in emergencies, tightens interconnection across departments and specialities [25], how the implementation of new technology can affect the working environment [26], challenges differential understandings and representations of health information pose for end-users and patient safety [27-29], and the strategies used by healthcare staff to achieve resilience and avoid conflicts under high-pressure conditions [30,31]. Researchers of joint cognitive systems have began to develop theories that incorporate a complexity perspective into the understanding of medical failure and success [23]. Such ideas present an opportunity for the exploration and development of the notion of crisis—empirically as well as theoretically building further on these ideas, a new focus of analysis for health care crisis can emerge from looking at the notion of escalation.

### Escalation

The process of escalation has previously been defined as a situation “where an initial irregularity develops into a continually deteriorating situation and starts affecting other areas in an accelerating tempo, with consequences that are difficult to overview and impossible to predict” [32] (p. 220). Woods and Patterson [33] outline that “the concept of escalation concerns a process – how situations move from canonical or textbook to non-routine to exceptional”. This position acknowledges the need for studying the dynamic process preceding the definition of a state of crisis.

Furthermore Woods characterises escalating situations in the following ways:

· A cascade of effects occurs in the monitored process.

· Demands for cognitive activity increase as the problem cascades.

· Demands for coordination increase as the problem cascades.

· The cascade and escalation represent a dynamic process.

These are important characteristics that should encourage further analysis. The first point is that by linking cascade of effects to a monitored process risks uncoupling this process from its social context. The next two points follow from the first. It assumes that cognitive activity and socio-cultural coordination increase as a result of the cascading problem rather than treat both cognitive activity and these coordination efforts as an inherent part of the escalating process. This in turn can further divorce the escalation process from its social context and this can lead to arbitrary analytic divisions say between inside and outside the organisation.

Using examples from maternity wards, our focus so far has been on the escalating labour situations starting of as normal, but at some point changing to be defined otherwise followed by the involvement of more staff from different wards, professions and hierarchies. Such situations can deteriorate and at some point a decision to perform an emergency Caesarean section might be taken. This is followed by the treatment of not one but two patients (the child and the mother). In previous work we have, based on naturalistic studies, suggested that seemingly simple intervention decisions (such as when a midwife calls a physician for help) can be interpreted to be complex to the point that the model of strict adherence to best practice guidelines needs to be complemented with one of enhancing organisational diversity [19]. In this debate article, we will argue that not only standard practices for how to manage escalating situations needs to be critically reflected upon, but also current best practice ways of understanding crisis and emergencies in healthcare need to be rethought. Instead of viewing escalation as a process uncoupled from its social context, the present analysis suggests that it is necessary to outline a theory for escalation as a social process in which coordination and cognitive activity are highly interrelated. In this sense our model of escalation is not simply one of organisational escalation or clinical escalation, in fact we questions whether any analytical choice to separate the two can be fruitful. Rather we see both as highly interrelated and consequently an analysis of escalation cannot separate the two.

## Discussion

Consistent with the principle of epistemological pluralism [34] this discussion will explore, using multiple theoretical perspectives, the problem of obstetrical interventions. Here these will be treated as partially overlapping and sometimes contradictory accounts generated around critical decision points. The intent here is to help develop a vocabulary and a scientific agenda that can help us better understand the social process of escalation and the role these event(s) plays in health care crisis.

### The contingency theory perspective

Organisational contingency theory, which attempts to describe the best way to organise human work given a particular set of environmental conditions, assumes that quickly changing local conditions can best be handled by decentralised organisations. Having central decision makers involved in all organisational actions and responses can create unacceptable delays and information bottlenecks [35]. Rather, advocators of the theory emphasise that the more dynamic the environment, the more organic and immediate the organisational structure may become. Contingency theory typically invokes the example of military organisations, which are extremely hierarchical in peacetime with a heavy emphasis on planning, standardisation and top down protocol like drills and ceremony, with the discipline and power necessary to enforce all these aspects of life. On the battlefield however, these structures not only become less rigid and more flexible in response to fluid and haphazard situations but also flatten, with fewer explicit appeals made to rank. There can even be rank inversions where lower-ranking personnel may be better positioned (and equipped with more local knowledge) to take appropriate action than higher-ranking officers [36].

These findings have been replicated in a number of studies of high-reliability organisations [37-39]. This suggests that the decentralisation of decision-making authority, especially in regard to safety issues, can permit rapid and appropriate responses to dangers by the people closest to the problems at hand [40]. Wildavksy called this “decentralised anticipation,” and argues that micro-level entrepreneurial efforts can do more to improve safety than any centralised and restrictive top-down policies or structures [41]. High reliability theory also stresses the need for operational discretion at lower levels in the organisation. Even the lowest ranks on the deck of an aircraft carrier have the authority to suspend any take off or landing that might pose unnecessary risk [42]. As a result, high reliability has looked carefully at roles validation of minority opinion [38] and encouragement of dissent [43] can play in averting crisis, something echoed in the recommendation to treat nurses as a primary investment in patient safety [44].

When it comes to obstetric interventions, however, contingency theory does not seem to hold water. Rather than become a flatter, more organic organisation, obstetrics (and healthcare in general) seems to do something quite different in crisis and emergencies. This is to make more use, not less, of expertise embedded in hierarchy and structure [45,46]. For example, one of the most important intervention decisions for hospital midwives is whether to call a resident (physician) for assistance.^b^ If this decision is made, the organisation seems to have added another layer in response to the escalating problem. Organisational changes occurring during health care escalations can be characterised as upward and outward “reach” to different levels and types of institutionalised hierarchy/competences. This seems to pick up tempo and pace if the situation deteriorates. During a crisis not only the charge of the patient is handed over to the physician, but also the control of the ward and the work carried out there at least in the “intermediate model” of labour care we studied. Midwifes essentially “step back” from the responsibility of the judgment characteristic of all aspects of normality [47]. Intervention decisions, in the terms of actively delivering the baby, and thus handling the crisis, then formally belong to the physician with the midwifes no longer seeing themselves as responsible, especially in any bureaucratic sense of the term, for whatever happens next [19].

This, from the point of view of contingency theory, seems counter intuitive. To understand situations like this we require new models and perspectives. In particular, what we do not understand very well is why hierarchy and structure (often invoked as lay and even “scientific” reasons for failure in crises and emergencies) sometimes function as very effective resources in healthcare crises. The literature studying healthcare professionals has largely focused on the relationship between novices and experts [48]. Some literature is also available on the models of competence and hierarchy that inform the health care system [49]. However, how expertise and authority “shift” during crisis is not something that the literature so far has not focused on.

### The explanatory model of incomplete interaction

The addition of further layers of organisation in escalating emergencies generates what the healthcare literature typically characterises as “communication problems”. The result is problems arising in emergencies and escalations are often treated as (or reduced to) a failure of communication [50,51]. To what extent this is an appropriation of a common sense discourse, one that equates communication disorders with institutional escalations or crises, it is too early to say. But note that what is at work here is a common fallacy derived from folk sociology: Namely if the individual or individuals act “correctly” the same will be true for the social institutions they work within.

This kind of thinking *Durkheim* years ago tried to correct still seems to underlie much of the literature on healthcare emergencies. Communication problems are typically the targets for safety intervention in healthcare [7-9,52] and it is difficult to know, given the present state of research, how positively these interventions have impacted. However to reduce problems of organisational choreography in an escalating crisis to simply a communication problem tends to “flatten out” perhaps prematurely the explanation curve. Other factors that might be at work here include institutionally mediated historical and temporal dimension (tempo) of these incidents. When factors like these are not acknowledged, there is a risk that other significant issues such as power and hierarchy are muted [53]. Unfortunately, in safety and organisational studies, the analysis and “solutions” that are often proposed to handle clinical error tend to finesse issues of this kind. What actually occurs is that these aspects (and the kinds of negotiated practices we have been discussing here) essentially “fall out” of most analyses of health care safety.

To explain escalation in terms of incomplete interactions between actors creates several analytic problems and resolutions. The first is the reduction of complex event chains and choreographies^c^ – ones that are seldom successfully handled by any linear model – to some kind of social atomism (or psychologism) in which a single actor is thought be to (and is often held) accountable for that particular crisis [54,55]. As well, the literature on healthcare and safety tends to valorise individual performance so both a “hero” is praised for his/her success, and/or individual actors are singled out for blame [56,57]. In fact, in most explanations of crises situation, there is a tendency not to “go above” the shop floor. This too reinforces the tendency in the literature, to provide to accounts of individual competence and failure. This of course says much about the value Westerners place on the individual and on the individual competence and autonomy. However this can mask the importance event chains and clinical chorography can have for healthcare institutions and clinical effectiveness.

### Escalation as a social process

Treating escalation as a process decoupled from the organisation, and one that can be managed by the best practice routines (such as calling for assistance at the point where the situation is no longer being normal), holds the risk of underestimating “the social construction of the context that both legitimates a particular form of action and constitutes the world in the progress … [so] we might begin to consider not what is the *situation*, but how it is *situated*” [58] (p. 1471). A simple example of how the process of *situating* constructs the obstetric *situation* could make use of the concept of *active management of labour*[59]. Here, midwifes are instructed not to accept patients to the delivery unit unless they are in active labour. The reasons for this are related to the conviction that labour is not a process that benefits from hospitalisation before labour has entered its “active phase”. Accepting patients “too” early in the natural process of labour can well medicalise problems a delivery unit cannot handle typically well. This can lead to a situation where a normal birth is defined as a non-normal labour (a definition that influences the social construction of escalating obstetrical situations) and the response is to medically induce labour. This can introduce considerable risk compared to the natural onset of labour. This construction and definition of context can also be a means to negotiate power (adding or not adding another layer of the hierarchy) and, every time it is applied, it also reinforces the medical hierarchy model.

In the healthcare literature, the shift of responsibility as a situation deteriorates could be written off as just a shift of responsibility required due to an increasingly more complex situation, to an actor with more knowledge (as the hierarchy-competence model does). However, when looked at closely, this explanation seems too facile. In fact almost all midwifes are well aware that they may often be calling someone with less experience and competence than themselves to handle crises or emergencies because in most deliveries one turns first a junior physician [19].

What these calls for an obstetrician do is in effect to mark an organisational transition, i.e., a shift from a normal situation to a crisis. In Foucault’s terms, these calls enable midwives to renegotiate “the order of things” [60,61], and this occurs through their redefinition (their release of control and authority) in a specific institutional “activity”. Midwifes can wield considerable power in the delivery room because they can construct and define situations there as normal or not. For a midwife virtually any kind of ward event can become evidence for one definition or another regarding the ward’s and patients’ status. Any number and variety of factors like the emergency staff on duty, number of patients at the ward, the length of pregnancy, or time of the day can be cited by midwives as evidence that a situation is normal or not.

When viewed this way, an organisation’s shift from normal state to crisis cannot simply be seen as a response to a deteriorating process. The example of the midwives here shows the need to see escalation as a social process – one constructed by those who can alter or improvise on on-going habitual practice sometimes even to the extent that it can throw normal practice into question. There is seemingly a paradox at work here. In healthcare crises, hierarchy, i.e., power can be invoked to redefine the situation so that alternatives to local practice emerge. At the same time, the redefinition of local practice can also confirm the logic and rationality of normal practice and hierarchy. It is this simultaneous, challenging, extending and re-legitimatising of traditional practice and hierarchy that occurs in almost any crisis. This helps explain why these events can be so refractory to most forms of social analysis. In other words, the structural and social processes at work in any escalation can be difficult to capture or analyse, especially given the standard models of health care work and health care organisations. Further, the construction of the contingencies (emphasised by the actors themselves) can mask the role that power and hierarchy play in the process. However, when we shift focus and view escalation as an inherently social process, the construction of events there can be seen, among other things, as an exercise of power - one that occurs in and as response to a complex social world.

Again, the kinds of organisational changes that can occur during an escalating situation in delivery units do not follow the patterns predicted by classical contingency theory. In health care for example, the response seems to be to further rely on (and build) hierarchical structures. However, analysing event chains, and the way these midwives first problematise and then legitimatise the handover of responsibility in a crisis can lead us closer to understanding how healthcare emergencies and crises get defined as such. In fact, it may be useful to trace out how hierarchical structures and local practice and resources mutually become redefined and extended during escalation process that precedes any crisis. This could have potentially more yield than for example continuing to focus on the role communication plays in crisis situations.

## Implications

Taking account of the social processes of escalation offers a new promising focus for healthcare research – one that has epistemological as well as practical/pragmatic implications. It is suggested that organisational choreography, involving an interplay of potential factors such as power, professional identity, organisational accountability, and experience, become a new focus for naturalistic action research [62]. Rather than seeking an objective “place to stand”, such studies of escalation emerge from and reflect the results of long term, on-going collaborations between practitioners and researchers. Consistent with action research’s best practice, what is identified as both “problem” and “solution” can be reformulated iteratively over the lifetime of the project. So far we have mainly studied the surface of *what* constitutes local practice in escalating situations. The next crucial question to answer is *why* the interpreted local practice is seen as a logical part of institutional practice. Questions to ask here include: Why is it perfectly logical for a senior midwife to call a much more junior physician to take over the responsibility of a non-normal situation? How does a crisis “deform” traditional, habitual, even historically determined local clinical practice, especially the ones practiced across and between clinical specialities? Clinical practice of course is local, as are all responses to emergency situations. But how does local practice change when crises occurs and escalates? It is this escalation, this shift in tempo, that sets off a crisis from normal events and this qualitative change is central for us to understand if we are to build an “anatomy of escalation” in much the same way as Plumb et al. argued for an “anatomy of patient safety” in a previous issue of this journal [63].

The more pragmatic implication of the research concerns the ways in which healthcare practitioners are now prepared for the complexities of escalating situations. Rather than preparing for crisis management by fostering “more appropriate” behaviour of individuals, the focus on escalation turns towards problems of interaction, coordination and enhancing organisational diversity [64]. In times when “production efficiency” is the most celebrated aim of the healthcare system (at least in the Scandinavian country that we have studied) just providing the possibility for midwives, obstetricians, anaesthesiology nurses, operation ward nurses, anaesthesiologists, paediatric nurses and paediatricians to meet and problematise issues concerning their interactions in the times that they become tightly coupled and highly interdependent, could be seen as a success. We hope to bring readers of this journal reports soon about the outcomes of such processes.

If we better understand not only how local practices are exploited in crisis but also how local practice can deform under stress, this could lead to innovation in both knowledge and practice. Using terms like escalation, event chains and choreography could help us strengthened the analysis of clinical acts and competence. This can help us turn our attention, as all good models should, to important elements in an important class of social phenomenon, crises and emergencies, that we so far have overlooked.

## End notes

^a^ The notion of coupling is here used much in the same way as in Snook’s analysis of a Friendly-fire accident over northern Iraq in 1994 [15]. Analysing how diverse actors adapted locally under years of self-governance what was locally rational behaviour in “normal” circumstances made the system fail in the “non-normal” situation when the actors became tightly coupled. The locally rational behaviour of all actors involved, according to Snook, made two US F15 pilots shoot down the own Army’s helicopters once the actors became tightly coupled and highly interdependent.

^b^ In the Scandinavian country that we studied, midwives are specialist nurses with a significant medical authority. Unless the situation deviates from what is considered “normal” pregnancies as well as labours are facilitated by specialist nurses (midwives) without involvement of physicians.

^c^ An event chain has much in common with genealogy “tree” but rather than trace family or inheritance, it enables us to track the “hand off” of resources, knowledge, and practice, within and between, that occur between different healthcare domains routinely and in emergencies. The term choreography refers to issues of tempo, pace and timing as well as the differential reliance, informed both historically and by the moment, to healthcare/clinical resources.

## Competing interests

The authors declare that they have no competing interests.

## Authors’ contributions

JB, PhD, has the social processes of escalation in health care settings as his main focus. He had the main responsibility of preparing the manuscript. IA-W, MD, PhD, being an obstetrician, provided the medical expertise in the study. She is also involved in data collection activities together with JB. JMN, PhD, and SD, PhD, both assisted in conducting the analysis. All the authors provided important contributions for the preparation of the manuscript. All authors read and approved the final manuscript.

## Pre-publication history

The pre-publication history for this paper can be accessed here:

http://www.biomedcentral.com/1472-6963/12/161/prepub
